# Role of Cholesterol and Lipid Rafts in Cancer Signaling: A Promising Therapeutic Opportunity?

**DOI:** 10.3389/fcell.2021.622908

**Published:** 2021-03-19

**Authors:** Rosa Vona, Elisabetta Iessi, Paola Matarrese

**Affiliations:** Center for Gender-Specific Medicine, Istituto Superiore di Sanità [Italian National Institute of Health], Rome, Italy

**Keywords:** cholesterol, cancer, lipid rafts, cell death, invasion, metastases, therapy

## Abstract

Cholesterol is a lipid molecule that plays an essential role in a number of biological processes, both physiological and pathological. It is an essential structural constituent of cell membranes, and it is fundamental for biosynthesis, integrity, and functions of biological membranes, including membrane trafficking and signaling. Moreover, cholesterol is the major lipid component of lipid rafts, a sort of lipid-based structures that regulate the assembly and functioning of numerous cell signaling pathways, including those related to cancer, such as tumor cell growth, adhesion, migration, invasion, and apoptosis. Considering the importance of cholesterol metabolism, its homeostasis is strictly regulated at every stage: import, synthesis, export, metabolism, and storage. The alterations of this homeostatic balance are known to be associated with cardiovascular diseases and atherosclerosis, but mounting evidence also connects these behaviors to increased cancer risks. Although there is conflicting evidence on the role of cholesterol in cancer development, most of the studies consistently suggest that a dysregulation of cholesterol homeostasis could lead to cancer development. This review aims to discuss the current understanding of cholesterol homeostasis in normal and cancerous cells, summarizing key findings from recent preclinical and clinical studies that have investigated the role of major players in cholesterol regulation and the organization of lipid rafts, which could represent promising therapeutic targets.

## Introduction

Cholesterol is a primary lipid molecule that plays an essential role in a number of biological processes, both at physiological and pathological level (Maxfield and Tabas, 2005).

**FIGURE 1:**
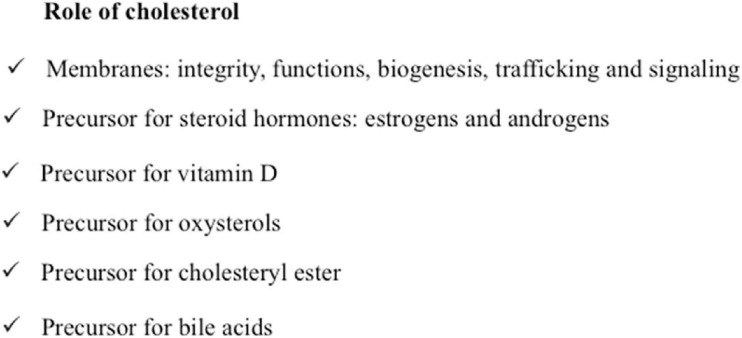
Principal functions of cholesterol. Figure inspired by Mollinedo and Gajate (2020).

Cholesterol, in addition to being an important constituent of cell membranes, is fundamental for their biogenesis, and is indispensable for maintaining integrity and functions of biological membranes, including endocytosis, membrane trafficking, and signaling (Maxfield and Tabas, 2005; Yamauchi and Rogers, 2018). Inside the cell, cholesterol, heterogeneously distributed among the organelles, modulates the immune system, and represents a precursor of hormones such as sexual hormones and vitamin D (Mollinedo and Gajate, 2020; Figure 1).

Recently, cholesterol has played a key role in cancer research because of its potential therapeutic implications both in prevention and in treatment. However, the role of cholesterol in oncogenesis is still debated (DuBroff and de Lorgeril, 2015). Literature data reported a contradictory role of cholesterol depending on the type of tumor (Ding et al., 2019). Excess of cholesterol is related to breast, colon, rectal, prostatic, and testicular cancers (Llaverias et al., 2011; Pelton et al., 2012; Murai, 2015; Radisauskas et al., 2016), while some prospective cohort studies showed an inverse association in gastric and prostate cancers (Asano et al., 2008; Heir et al., 2016). This review aims to discuss the current knowledge of cholesterol homeostasis, critically analyzing the most recent preclinical and clinical studies investigating the role of the principal players of the cholesterol biosynthetic pathway, and of the cholesterol-based membrane structure’s lipid rafts in the field of cancer.

## Cholesterol Metabolism

**FIGURE 2:**
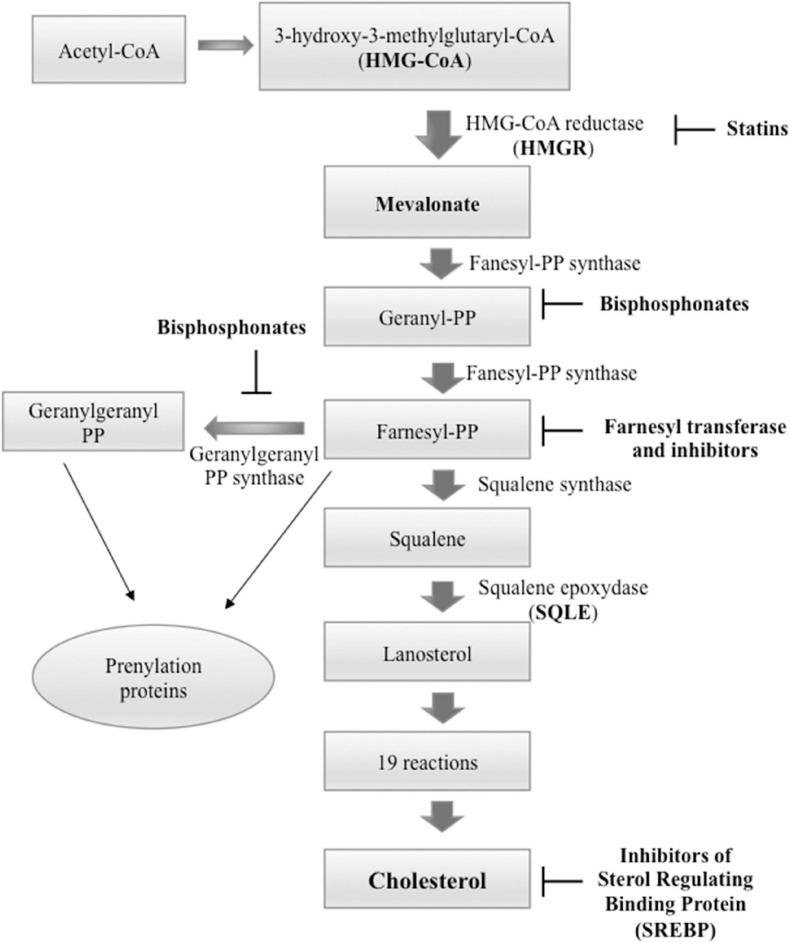
Cholesterol biosynthesis pathway and principal inhibitors. Starting from three molecules of acetyl-coenzyme A (CoA), cholesterol is synthesized in more than 20 enzymatic steps. 3-Hydroxy-3-methylglutaryl-CoAreductase (HMGCR) and squalene epoxidase (SQLE) act as rate-limiting enzymes. The principal inhibitors of cholesterol biosynthesis are statins that inhibit HMGCR, inhibitors of sterol regulating binding protein (SREBP) that inactivate the transcription of cholesterol biosynthesis genes; and bisphosphonates that act downstream of statins and inhibit farnesyl pyrophosphate synthase with consecutive decrease of the farnesyl pyrophosphate and geranylgeranyl pyrophosphate. This step of the cholesterol biosynthesis is also targeted by farnesyl transferase inhibitors.

Cholesterol is produced through a cascade of enzymatic reactions, namely, mevalonate pathway, which requires the participation of different enzymes localized on the membranes of the endoplasmic reticulum (ER). Briefly, the combination of three acetyl-CoA molecules leads to the formation of one 3-hydroxy-3-methylglutaryl coenzyme A (HMG-CoA) molecule. The latter, by the action of HMG-CoA reductase (HMGCR), is converted into mevalonate, in turn transformed into squalene (SQL), and ultimately into cholesterol by means of a number of reactions (Figure 2). Food can be a source of cholesterol too. In fact, the Niemann–Pick type C1-like 1 (NPC1L1) protein, present on the membrane of the intestinal enterocytes, is responsible for the absorption of cholesterol, which is released as chylomicrons, triglyceride-rich lipid particles, and is taken up by the liver (Altmann et al., 2004; Luo et al., 2020).

Cholesterol is mainly synthesized in the liver and free into the bloodstream as very-low-density lipoproteins (VLDLs). In the bloodstream, the VLDLs are transformed to produce low-density lipoproteins (LDLs), transported to peripheral cells by the bloodstream (Ikonen, 2008; Goldstein and Brown, 2009). LDLs enter cells via receptor (LDLR)-mediated endocytosis and are transported to lysosomes where they are hydrolyzed into free cholesterol molecules, which are transported to cellular membranes to carry out its multiple functions (Brown and Goldstein, 1986; Ikonen, 2008; Maxfield and van Meer, 2010; Kuzu et al., 2016).

Mevalonate pathway is tightly regulated by transcriptional and translational mechanisms capable of responding to physiological signals. Cholesterol biosynthesis is regulated by four principal players: (1) sterol regulatory element-binding protein 2 (SREBP2), which acts through a negative feedback mechanism (Sato, 2010), (2) liver X Receptors (LXRs), (3) HMGCR, and (4) squalene epoxidase (SQLE). HMGCR and SQLE are rate-limiting enzymes, which can regulate cholesterol biosynthesis, the reactions they catalyze being energetically expensive. When intracellular ATP levels are low, 5′adenosine monophosphate-activated protein kinase (AMPK) phosphorylates HMGCR inhibiting its function (Loh et al., 2019). Moreover, HMGCR is influenced by the presence of LDL in the medium; in fact, upon LDL starvation, HMGCR activity enhances, while it highly decreases when LDL is added back. On the other hand, once cholesterol has exhausted its function, its surplus is exported via ATP-binding cassette (ABC) subfamily A member 1 (ABCA1) or ABC subfamily G member 1 (ABCG1) to lipid-poor apolipoprotein A-I (ApoA-I), thus generating high-density lipoproteins (HDLs) (Gelissen et al., 2006; Lorenzi et al., 2008; Daniil et al., 2013; Phillips, 2014). The transcription of ABCA1 is upregulated by nuclear LXRs when the intracellular cholesterol level is high (Wang et al., 2008; Ouvrier et al., 2009; Kuzu et al., 2016). The CoA:cholesteryl acyltransferase 1 (ACAT1) transforms excess cholesterol into less toxic compounds, such as cholesteryl esters (CEs), which are stored as lipid droplets and used for the production of the main plasma lipoproteins (chylomicrons, VLDLs, LDLs, and HDLs). HDLs are then transported from peripheral tissues back to the liver and intestine, to recycle or eliminate cholesterol, and to steroidogenic organs, where cholesterol is used to generate steroid hormone (Chang et al., 2009; Luo et al., 2020).

Recent studies on microRNAs (miRNAs), a class of non-coding RNAs, highlighted their role in the cholesterol homeostasis by adjusting some important components of the system (Wagschal et al., 2015). For instance, under low sterol concentration conditions, higher transcription of miR-33a is required to control cholesterol export and HDL metabolism through ABCA1 inhibition (Wagschal et al., 2015). Conversely, miR-223 regulates cholesterol amount by inhibiting its production and ameliorating cholesterol efflux by enhancing ABCA1 levels (Vickers et al., 2014). miRNA-122, present mainly in hepatocytes, when inhibited significantly suppresses blood cholesterol level (Rotllan and Fernandez-Hernando, 2012). miR-27a has been shown to control HMGCR level either by a posttranslational block or by mRNA degradation (Khan et al., 2020). Strikingly, other miRNAs have been discovered by meta-analyses to be associated with cholesterol–lipoprotein trafficking alterations, such as miR-128-1, miR-148a, miR-130b, and miR-301b. These miRNAs were able to increase circulating cholesterol by controlling the expression level of LDLR and ABCA1 (Wagschal et al., 2015).

These data suggest the participation of miRNAs in the control of cholesterol metabolism, underlining how they could contribute to the alteration of cholesterol levels when dysregulated.

### Cholesterol Homeostasis in Normal and Cancer Cells

Considering the importance of cholesterol metabolism, its cellular homeostasis is strictly regulated at every stage: import, synthesis, export, transport, and esterification (Ikonen, 2008). Sterol regulatory element-binding protein 2 (SREBF2) and LXRs act as key regulators of cholesterol homeostasis (Ikonen, 2008). In the ER, cholesterol itself regulates its homeostasis. Low cholesterol levels induce translocation of SREBP2 to the nucleus where it promotes activation of genes implicated in the biosynthesis (e.g., HMGCR) and uptake (e.g., LDLR) of cholesterol (Ikonen, 2008). High cholesterol levels inhibit the cholesterol synthesis and facilitate its export through the activation of LXRs by oxysterols, oxidized derivatives of cholesterol (Wang et al., 2008; Kuzu et al., 2016). Recent studies on LNCaP prostate cancer cells highlighted an important protective role of LXRs (Pommier et al., 2010; Fu et al., 2014). In fact, the activation of these transcription factors regulating cholesterol homeostasis was able to induce cell cycle arrest and to promote apoptosis (Pommier et al., 2010). The relationship among LXRs, cholesterol, and prostate cancer highlights that LXRs have good chance to be targeted one day in this tumor.

During cholesterol biosynthesis, depending on the type of tissue, different intermediate sterols are formed, such as cholesteryl ester, oxysterols, bile acids, cholecalciferol/vitamin D, and various steroid hormones. All these sterols have important physiological functions in cells and in tissues (Simons and Ikonen, 2000). Some cholesterol metabolites may also contribute to the progression and metastatization of some types of cancer (Lin et al., 2013; McDonnell et al., 2014; Baek et al., 2017).

**FIGURE 3:**
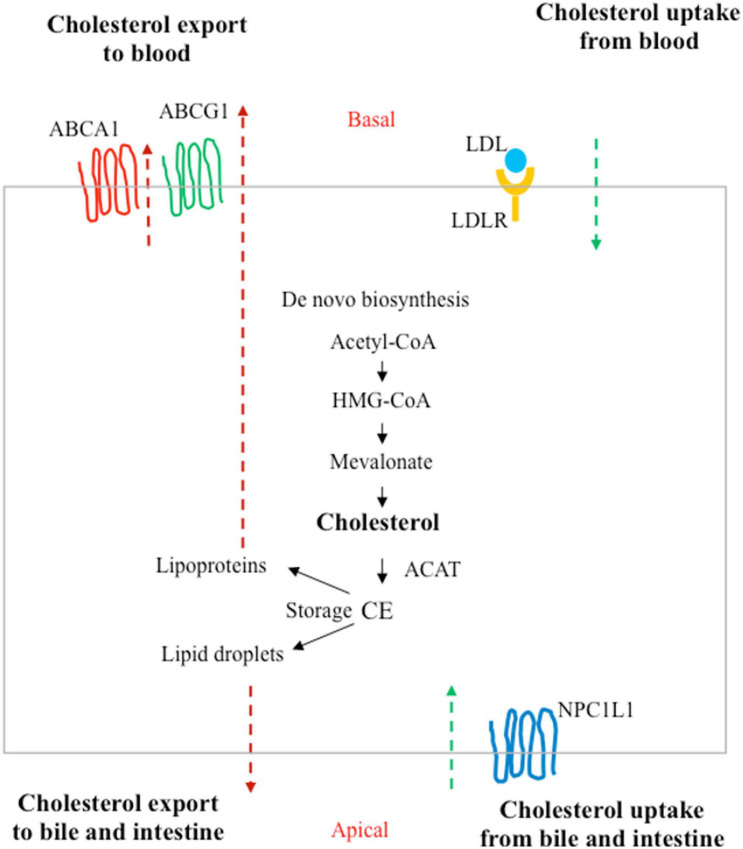
Homeostasis of cholesterol in a polarized cell. In addition to *de novo* biosynthesis, cholesterol carried by low-density lipoprotein (LDLs) in the blood can be taken up by LDL receptors (LDLRs) at the basal surface of the polarized cells (such as enterocytes or hepatocytes). Niemann–Pick type C1-like 1 (NPC1L1) can absorb free cholesterol from dietary sources by enterocytes in the intestine and from bile in the biliary ducts by hepatocytes in the liver. Excess cholesterol is exported to the blood by ATP-binding cassette subfamily A member 1 (ABCA1) or the subfamily G member 1 (ABCG1). Cholesterol can also be converted to cholesteryl ester (CE) by acyl coenzyme A:cholesterol acyltransferase (ACAT) for storage in lipid droplets or for secretion as lipoproteins.

Under healthy conditions, the amount of cholesterol is the result of a balance between synthesis, uptake from extracellular milieu, removal of the cholesterol surplus from peripheral tissues, and metabolic conversion (Simons and Ikonen, 2000; Figure 3). The alterations of this balance, often caused by unbalanced diets and unhealthy lifestyles (Hu et al., 2012), are known to be associated with cardiovascular diseases and atherosclerosis, but mounting evidence also connects these behaviors to increased cancer risks (Luo et al., 2020).

Over the past few decades, literature data have demonstrated that metabolic alterations represent a hallmark of cancer. Oncogenesis is an intricate process that involves, in addition to reprogramming energy metabolism, also reprogramming of genetic information, signaling mechanisms, and structural components, which are critical for the survival and growth of cancer cells (Hanahan and Weinberg, 2000, 2011). Reprogramming energy metabolism had already been described by Otto Warburg, which highlighted that cancer cells, even in the presence of abundant oxygen, preferably produce ATP (adenosine triphosphate) through the less efficient glycolytic pathway, rather than through oxidative phosphorylation (OXPHOS), thus producing more lactate than normal cells (Warburg, 1925). This metabolic switch of cancer cells, called Warburg effect, is also known as aerobic glycolysis. More recently, attention has been paid to metabolic heterogeneity within the tumor. Particular emphasis has been given to the cells present in the tumor stroma, which would also have the role of metabolically supporting the cancer cells. In this context fits the theory of the reverse Warburg effect according to which the aerobic glycolysis in the cells of the tumor stroma would have the role of providing, in addition to ATP, metabolites for the generation of further ATP through OXPHOS in cancer cells. Moreover, the metabolic coupling between stromal and tumor cell, with the exchange of useful metabolites, would also allow the tumor cell to increase its proliferation and reduce cell death (Xu et al., 2015; Wilde et al., 2017; Benny et al., 2020).

**FIGURE 4:**
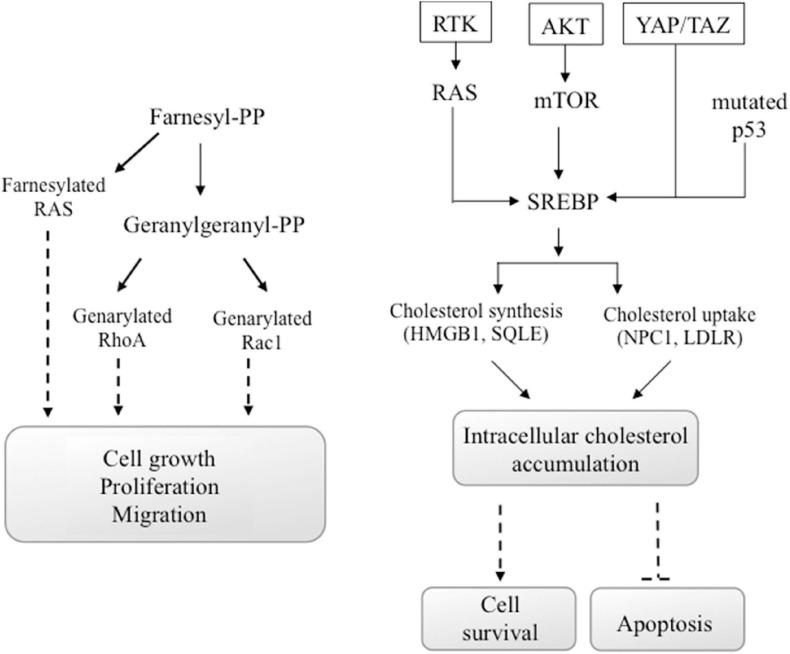
Oncogenic signals involving cholesterol. Some oncogenic signals initiated from AKT/mTOR, RTK/RAS, YAP/TAZ, or mutated p53 induce the activity of SREBP transcription factor, the major regulator of genes encoding cholesterol synthesis and uptake. Also, the activation of the GTP-binding proteins Ras, Rho, and Rac, which functions are involved in carcinogenesis, depends on the availability of cholesterol. In particular, Ras family proteins are prenylated by farnesyl-PP, whereas geranylgeranyl-PP prenylates proteins of the Rho and Rac families. These chemical modifications are mandatory for their activity.

Alteration of energy metabolism is due to enhanced metabolic needs of cancer cells to support the proliferation, migration, and metastatic cancer activities (Warburg, 1956; Silvente-Poirot and Poirot, 2012). However, there are conflicting epidemiological evidence on the cholesterol function in cancer development, while important preclinical data suggested that a dysregulation of cholesterol homeostasis could lead to cancer progression. In particular, new data suggested that cholesterol amount in cells may have a greater role in cancer development than serum cholesterol (Kuzu et al., 2016). Some oncogenic pathways such as PI3K/AKT/mTOR, RTK/RAS, YAP/TAZ, and p53 are also able to regulate cholesterol production in cancer cells (Figure 4).

## Cholesterol as Component of Lipid Rafts

Besides the classical role of cholesterol in determining the correct structure, fluidity, and functioning of eukaryotic cell membranes, cholesterol represents the prevalent lipid component of specific plasma membrane microdomains, known as lipid rafts, also contributing to their organization. Indeed, cholesterol accumulates in specialized region of the membrane and, by interacting with sphingolipids, forms lipid rafts (Brown and London, 2000; Pike, 2003, 2006). These membrane regions are heterogeneous and highly dynamic structures, ranging from 10 to 200 nm in size, that selectively recruit and concentrate different membrane proteins (i.e., receptors, adhesion molecules, etc.) and signaling molecules, forming a sort of platform for signal transduction (Simons and Toomre, 2000). Nitric oxide synthase, GPI-anchored proteins, Src-family tyrosine kinases, G-protein-α subunit, protein kinase C, and protein kinase A are typically localized within lipid rafts (Song et al., 1997; Galbiati et al., 1999; Razani et al., 1999; Patel and Insel, 2009).

Membrane and signaling proteins reside transiently within lipid rafts. Thus, depending on whether the signal transduction needs to be enhanced or damped, they can be recruited or excluded from lipid rafts in a reversible manner. The mechanisms at the base of reversible raft–protein associations are not yet completely understood. Characteristic lipid post-translational modifications have been described by researchers to be responsible for the recruitment of proteins within lipid rafts. One of the major raft-targeting signals is represented by S-palmitoylation of transmembrane proteins, consisting in palmitate moieties added to cysteine residues through thioester linkage (Resh, 1999, 2004; Petrova et al., 2013). For instance, death receptors such as Fas/CD95 (Chakrabandhu et al., 2007; Feig et al., 2007) and TRAIL receptor 1 (Rossin et al., 2009) are palmitoylated on specific cysteine residues, and this event is essential either for their recruitment within lipid rafts or for an efficient transmission of death signals. In human breast cancer cells, palmitoylation of the surface adhesion receptor CD44 relies on two cysteine residues. This event enhances CD44 affinity for cholesterol-rich lipid rafts, inducing CD44 recruitment into lipid microdomains (Babina et al., 2014), limiting breast cancer cell migration (Donatello et al., 2012). Moreover, proteins can be anchored to plasma membranes through a glycosylphosphatidylinositol (GPI) anchor or they can present a lysine-rich region within their amino acid sequence, which represents an additional signal beside palmitoylation targeting proteins within lipid rafts (Rossin et al., 2009).

Therefore, membrane lipid rafts represent a sort of lipid-based structures that regulate the assembly and functioning of numerous cell signaling, including those related to cancer (Staubach and Hanisch, 2011; Donatello et al., 2012), such as tumor cell growth, adhesion, migration, invasion, and apoptosis (Yang et al., 2014, 2018).

Lipid rafts were also found in mitochondria, ER, in the nuclear membrane, and phagosomes, respectively, where they form a sort of “raft-like microdomain” (Garofalo et al., 2005, 2016; Brunham et al., 2006; Cascianelli et al., 2008; Boslem et al., 2013; Matarrese et al., 2014). These regions, rich in gangliosides, present a content of cholesterol lower as compared with plasma membrane (Garofalo et al., 2015). They are involved in triggering specific events during apoptosis execution, i.e., mitochondria hyperpolarization and depolarization, with consequent release of apoptogenic factors (Garofalo et al., 2005; Scorrano, 2008; Sorice et al., 2009), and regulation of fusion–fission processes (Garofalo et al., 2007; Scorrano, 2008; Sorice et al., 2009; Ciarlo et al., 2010, 2018; Annunziata et al., 2018).

Taking into account all these considerations, the interest in targeting membrane raft cholesterol in cancer cells is increasing. Specific studies might be helpful to better understand the potential use of lipid rafts as innovative target in anti-cancer therapy.

### Raft-Associated Cholesterol Involvement in Migration, Invasion, and Metastatic Processes of Cancer Cells

Mounting evidence of the literature supports a role for lipid rafts in several signaling transduction pathway related to malignancy. Therefore, the interest in studying lipid rafts and the modulation of lipid raft organization is growing. Approaches aimed at disorganizing membrane raft domains were reported by researchers to affect cancer cell proliferation, adhesion, migration, invasiveness, metastatic spread, and apoptosis (Simons and Toomre, 2000; Simons and Ehehalt, 2002; Zidovetzki and Levitan, 2007).

A critical feature of malignancy is represented by the acquisition of a more aggressive phenotype by cancer cells. Because of their malignancy, cancer cells became able to adhere to the extracellular matrix and therefore to migrate and to invade other tissues, leading to metastasis. The plasma membrane certainly represents the first structure of the tumor cell to be involved in the processes of invasion and metastasis. Invadopodia are highly specialized plasma membrane structures of tumor cells, corresponding to podosomes of untransformed cells, able to favor the adhesion and penetration of the tumor cell into the underlying extracellular matrix, stroma, and basement membrane. The formation of invadopodia, structures rich in cytoskeletal elements, adhesion molecules, and degradative enzymes, requires the presence of lipid rafts, which are decisive for the establishment of this invasive and dynamic structure (Nicolson, 2015). Yamaguchi et al. (2009) found that Cav-1, a typical component of caveolar membrane lipid raft structures, represents an essential regulator of invadopodia-mediated human breast cancer cell invasion (Yamaguchi et al., 2009). Disruption of lipid rafts by depletion of membrane cholesterol using methyl-β-cyclodextrin (MβCD) suppressed either invadopodia formation and tumor invasion, thus providing evidence that lipid raft formation was mandatory for invadopodia functioning, at least in human breast cancer (Yang et al., 2016). In bladder cancer cells, Cav-1 overexpression induced epithelial-to-mesenchymal transition that was mediated by PI3K/AKT activation (Liang et al., 2014).

Very interestingly, a reduced expression of Cav-1 was found frequently in human cancer-associated fibroblasts (Martinez-Outschoorn et al., 2014), where it was accompanied by a reduced mitochondrial function and an increased expression of glycolytic enzymes (Asterholm et al., 2012). Some studies have shown that tumor cells, inducing oxidative stress, were responsible for the autophagic degradation of Cav-1 in stromal fibroblast, thus favoring tumor–stroma co-evolution underlying the theory of the reverse Warburg effect (Martinez-Outschoorn et al., 2010).

The dysregulation of flotillin-2, a protein marker of non-caveolar plasma membrane microdomains, has been reported in a variety of tumors. In particular, a recent study related a high expression of flotillin-2 to the incidence of lymph node metastasis and TNM stage in intrahepatic cholangiocarcinoma cell lines (Xu et al., 2020), as also previously observed in a variety of human solid tumors (Liu et al., 2017). High expression of flotillins was also observed in drug-resistant strains of colorectal cancer cells (Ye et al., 2019). Data from Ye and coworkers clearly demonstrated that flotillin silencing using lentivirus-mediated RNAi approach, by inducing destruction of lipid rafts, inhibited the drug resistance of the colorectal cancer cell line HCT-15 (Ye et al., 2019).

Additional findings in prostate cancer cells suggested a potential positive role of membrane cholesterol in modulating the IL-6/STAT3 pathway and chemoattraction (Nakashima et al., 2000; Kim et al., 2004; Akashi et al., 2008; Chinni et al., 2008). The IL6–JAK–STAT3 pathway, known to be an important signaling pathway in prostate cancer, requires intact lipid rafts for sustaining pro-oncogenic signaling. Also in this case, disruption of lipid rafts by MβCD inhibited this pathway thus controlling prostate cancer growth (Dambal et al., 2020). In addition, it has also been reported that hypoxia, a prognostic predictor of poor clinical outcome (Vaupel et al., 2001), was able to increase cholesterol level and recruitment of Notch3 into lipid rafts. Notch3 activity sustained cell proliferation of different hormone-dependent and independent prostate cancer cell lines, and positively correlated with tumor aggressiveness (Danza et al., 2013). In prostate cancer cells, it has also been reported that the interaction of the tetraspanin CD82 with cholesterol in lipid rafts inhibited tumor cell movement and invasion by acting on plasma membrane/cytoskeleton linker ezrin, thus disengaging the plasma membrane–cytoskeleton connection and consequently repressing cell movement (Huang et al., 2020).

Several proteins responsible for development of a more aggressive and metastatic phenotype have been found associated with lipid rafts, including integrins and cell adhesion molecules, ion channels, potassium channels, and MUC1 (Staubach et al., 2009; Haddon and Hugh, 2015), strongly supporting a positive role for lipid rafts in tumor progression.

Several findings reported the localization within lipid rafts of the highly expressed cell surface adhesion receptor CD44, whose lipid raft localization was related to invasion and metastatic processes of cancer cells (Murai, 2015; Mollinedo and Gajate, 2020). In this context, it was reported that MβCD-mediated depletion of cholesterol induced CD44 shedding (Adler, 2011; Murai et al., 2011). Like MβCD, simvastatin, an agent that is able to target cholesterol with consequent disorganization of membrane rafts, also demonstrated to be effective in increasing shedding of CD44 and concomitantly in inhibiting tumor cell migration (Murai et al., 2004, 2006, 2009; Murai, 2015). In the same vein, it has also been shown that other statins induced disruption of lipid rafts, leading to impairment of tumor cell adhesion and migration (Murai et al., 2011; Murai, 2012).

Like other receptors, CD44 has been found into lipid rafts (Murai, 2015) or associated with the actin cytoskeleton through the ezrin protein in non-raft compartments (Martin et al., 2003; Yang et al., 2018). Once CD44 binds ezrin in non-raft compartments, it promotes cancer cell adhesion and migration in hepatocellular carcinoma cells (Yang et al., 2003). Similar results were also reported for breast cancer cells (Donatello et al., 2012). Thus, CD44–ezrin interaction in non-raft compartments leads to cell migration (Martin et al., 2003; Mollinedo and Gajate, 2020). Taken together, these data point out the importance of lipid rafts in CD44-mediated migration and invasion processes, but the molecular mechanism involved remains to be fully elucidated. Taking into account all these evidences, lipid rafts might therefore represent an important modulator of the invasive and metastatic ability of cancer cells. Accordingly, it has demonstrated that suppressing lipid raft formation using the bioactive natural compound daphnane-type diterpenes from *Daphne genkwa* (GD) strongly impaired human hepatocellular carcinoma cell invasion and migration (Wu et al., 2020).

### Raft-Associated Cholesterol in Cancer Cell Survival and Apoptosis

Many studies in the literature evidenced cholesterol-rich rafts as important players regarding cancer cell survival signaling and proliferative pathway. Indeed, it has been reported that survival signal pathways are compartmentalized into lipid rafts where membrane receptors can become in close contact with downstream signaling molecules, inducing the transmission of proliferative signals into the cells. This is the case of the activation of PI3K/AKT-mediated survival signals induced by insulin-like growth factor (Gao and Zhang, 2008, 2009; Gao et al., 2011; Reis-Sobreiro et al., 2013; Mollinedo and Gajate, 2020). In fact, it has been reported that activation of IGF receptors induced the recruitment of several signaling molecules involved in the PI3K/AKT pathway into lipid rafts, favoring activation by phosphorylation of AKT, with subsequent transmission of survival signals into the cells. Disruption of lipid rafts using MβCD or filipin III had demonstrated efficacy in inhibiting the activation of AKT and other survival signaling pathways in human lung adenocarcinoma and human T-cell leukemia, in favor of apoptosis activation (Motoyama et al., 2009).

Data obtained in prostate cancer highlighted the importance of the epidermal growth factor (EGF) receptor recruitment into lipid rafts for an efficient transmission of survival signal within the cells (Zhuang et al., 2002; Zidovetzki and Levitan, 2007). Indeed, it has been reported that disruption of membrane rafts using statins, known cholesterol-lowering agents, impaired EGF receptor pathway, favoring apoptosis (Zhuang et al., 2005; Oh et al., 2007; Hryniewicz-Jankowska et al., 2019).

**FIGURE 5:**
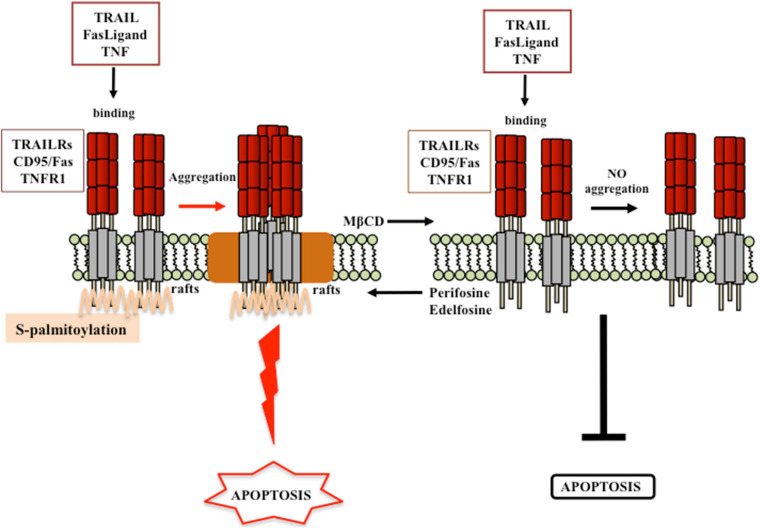
Proposed model for lipid rafts involvement in receptor-mediated cell death. According to the model proposed by Mollinedo and Gajate (2020), engagement of membrane death receptors within lipid rafts promotes their aggregation and the subsequent activation of cell death signaling pathway. Destruction of lipid rafts by cholesterol lowering (e.g., MβCD treatment) inhibits cancer cell death. By contrast, favoring the recruitment of death receptor within lipid rafts (e.g., perifosine or edelfosine treatment), the activation of death signals is favored. Modified from Gajate and Mollinedo (2015a,b); Mollinedo and Gajate (2020).

Particular attention has also been paid by researchers on the role of lipid rafts regarding the apoptotic signaling pathway, which could occur following one of the two classical pathways, the extrinsic membrane receptor-mediated or the intrinsic mitochondria-involving pathway. Interestingly, cholesterol has emerged as key modulator of both apoptotic pathways. Like for cancer survival signaling, lipid rafts have been described as scaffold plasma membrane regions, in which death receptors and downstream signaling molecules (FADD, pro-caspase-8/-10) aggregate and cluster in, favoring apoptosis induction and execution (Gajate and Mollinedo, 2001; Scheel-Toellner et al., 2002; Garofalo et al., 2003; Gajate et al., 2004; Malorni et al., 2007; Mollinedo and Gajate, 2010, 2020; Iessi et al., 2020). Findings from the last two decades evidenced that the localization of death receptors (e.g., CD95/FAS and TRAIL) into these specialized regions is mandatory either for susceptibility to receptor-mediated death signal or for a correct and efficient initiation of the apoptotic signaling (Figure 5; Gajate and Mollinedo, 2001, 2007; Mollinedo et al., 2004; Mollinedo and Gajate, 2006, 2020; Marconi et al., 2013). Indeed, recruitment of the death receptor Fas/CD95 into lipid rafts has been described in several studies (Gajate and Mollinedo, 2001, 2015a; Gajate et al., 2004; Mollinedo and Gajate, 2020). Similarly, TRAIL death receptor 1, but not TRAIL death receptor 2, localized constitutively within the lipid rafts in human cancer B-cell lines, and this event was depicted as mandatory for an efficient death receptor-mediated signaling transduction (Marconi et al., 2013). Actin cytoskeleton plays a peculiar role in ensuring a correct lipid localization of membrane proteins and an efficient cell death signal transmission (Algeciras-Schimnich et al., 2002; Gajate and Mollinedo, 2005). The ERM protein ezrin, known linker between membrane proteins and the actin cytoskeleton, was described as a positive regulator of early steps of Fas receptor signaling in lymphoid T cells (Parlato et al., 2000; Lozupone et al., 2004). Moreover, aggregation of death receptors within lipid rafts, by promoting apoptosis induction, was favored by several agents, i.e., oxaliplatin (Xu et al., 2009), epirubicin (Xu et al., 2011), β-elemene (Xu et al., 2018), bufalin (Yan et al., 2014), resveratrol (Delmas et al., 2004; Reis-Sobreiro et al., 2009), ursodeoxycholic acid (Lim et al., 2011), edelfosine (Gajate and Mollinedo, 2001; Gajate et al., 2004; Lim et al., 2016; Mollinedo and Gajate, 2020), and perifosine (Gajate and Mollinedo, 2007; Mollinedo and Gajate, 2020). On the contrary, agents able to disaggregate lipid rafts through membrane cholesterol depletion induced resistance to apoptotic cell death (Delmas et al., 2004; Gajate and Mollinedo, 2007, 2014, 2015b; Gajate et al., 2009; Marconi et al., 2013). For instance, resistance to TRAIL may be promoted by MβCD (Delmas et al., 2004; Marconi et al., 2013), whereas enhancement of TRAIL-induced apoptosis could be observed following perifosine treatment, which favored the recruitment of DRs into lipid rafts (Gajate and Mollinedo, 2007; Marconi et al., 2013). Edelfosine-mediated upregulation of TRAIL-R2, and its subsequent increased recruitment within lipid rafts, turned resistant gastric cancer cells back to a TRAIL-sensitive phenotype (Lim et al., 2016). Enhanced Fas/CD95 recruitment and clustering within lipid rafts, and consequent increased Fas/CD95-mediated apoptosis, was observed after edelfosine treatment in several hematological malignancies (Gajate and Mollinedo, 2001, 2007; Gajate et al., 2004, 2009; Mollinedo and Gajate, 2006, 2015; Mollinedo et al., 2010a, b). As a consequence, depletion of membrane cholesterol by leading to disruption of lipid rafts strongly impaired Fas/CD95 death signaling (Gajate and Mollinedo, 2007, 2014, 2015b; Gajate et al., 2009).

## Targeting Cholesterol Pathways as Cancer Therapy

Cancer has often been coupled with modifications in lipid metabolism, in particular in cholesterol metabolism. Alterations in cholesterol homeostasis have been identified in a large number of cancers. Both circulating and membrane associated cholesterol would appear to play a key role in the regulation of all cancer signaling pathways.

According with this, it has been suggested that several anticancer drugs may have anti-proliferative function, limiting the content or production of cholesterol. For instance, it has been shown that doxorubicin caused the death of cancer cells by promoting a reduction in HMGCR levels and causing a decrease in cholesterol content (Yun et al., 2019). Other data displayed that tamoxifen modulated cholesterol metabolism in breast cancer cells (Segala et al., 2013). Many natural compounds, including terpenoids, green tea, garlic extract, and curcumin, effective in cancer prevention and therapy, were identified to target cholesterol homeostasis in cancer cells (Mok and Lee, 2020). Thus, these natural compounds might be useful in regulating cholesterol homeostasis as adjuvants to complement current anticancer therapies.

**FIGURE 6:**
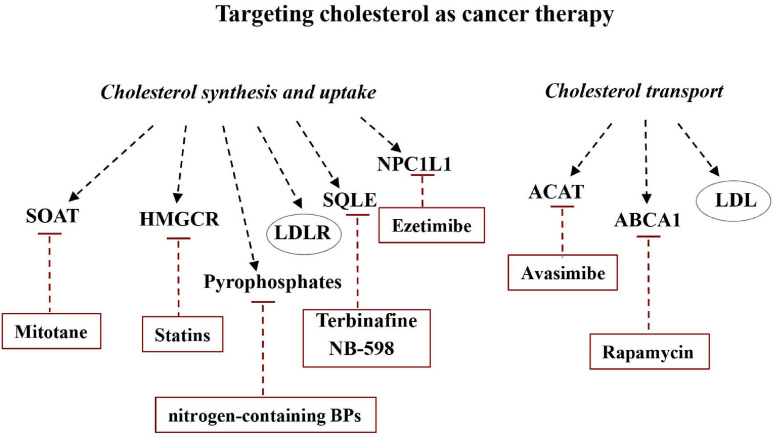
Targeting cholesterol as cancer therapy. Altering the balance between *de novo* synthesis of cholesterol and its transport could be useful for modulating cancer development. Some drugs or molecules are able to specifically inhibit some of the major players in cholesterol synthesis, uptake, and transport and can therefore be considered potentially useful in cancer therapy.

The cholesterol synthesis pathway consists of a number of enzymes, each of which can represent a possible target to disturb the mevalonate pathway in cancer cells. Many studies suggested that pharmacological modulation of intracellular cholesterol balance could be effective in controlling cancer progression (Figure 6).

### Targeting Cholesterol Synthesis and Uptake

Cellular cholesterol levels are the result of a balance among the processes of production, transport, and storage. The biochemical events and the main players that in cells are underlying synthesis and trafficking cholesterol, and which, therefore, can represent potential therapeutic targets, are briefly mentioned in the following section.

#### HMGCR

The rate-limiting reduction of HMG-CoA to mevalonate is an important regulatory step in cholesterol synthesis. HMG-CoA is highly regulated at transcriptional, translational, and post-translational planes (Goldstein and Brown, 1990). The HMGCR is an ER-localized glycoprotein that converts HMG-CoA to mevalonate. HMGCR is genetically activated by nSREBP2 when sterol concentrations are low. Sterols, mainly oxysterols, and two members of the vitamin E family can induce its degradation (Song et al., 2005; Chen et al., 2019). Moreover, ubiquitination and proteasome-mediated degradation are also known mechanisms regulating HMGCR. HMGCR changes from a phosphorylated to a dephosphorylated form. Phosphorylation inhibits HMGCR activity. AMP-activated protein kinase (AMPK) is the main actor for hepatic HMGCR phosphorylation, which in turn results in hampering of cholesterol production when low intracellular ATP levels are perceived. Conversely, HMGCR-mediated cholesterol biosynthesis is triggered when AMPK suppression occurs (Zhang et al., 2015; Luo et al., 2017; Soto-Acosta et al., 2017). The interaction between HMGCR and the insulin-induced gene protein (INSIG-1 or -2) at ER membrane is pivotal to HMGCR ubiquitination and degradation (Gong et al., 2006). Overexpression of HMGCR has been well documented in gastric cancer cells, when it promoted growth and migration, while HMGCR knockdown inhibited growth, migration, and tumorigenesis (Chushi et al., 2016; Yang et al., 2020). An upregulation of HMGCR was also observed in glioblastoma and prostate cancer (Qiu et al., 2016; Longo et al., 2019).

Statins were first used in the treatment of dyslipidemic disorders as therapeutic target to limiting production of mevalonate. In fact, statins are competitive inhibitors of HMGCR, and act by inhibiting the mevalonate pathway and reducing the end-product amounts such as cholesterol, isoprenoids, ubiquinone, and isopentenyladenine (Clendening et al., 2010; Thurnher et al., 2012; Cruz et al., 2013). Recently, a role in anti-cancer treatments has been proposed for statins (Farwell et al., 2008). Literature data showed how the use of statins, alone or in combination with chemotherapeutic agents, could have anti-tumor effects and reduce the onset of multidrug resistance (Mollinedo et al., 2010a; Cruz et al., 2013; Warita et al., 2014; Kuzu et al., 2016). As they have been shown very effective against mesenchymal-like cancer cells, Warita et al. have hypothesized their possible use during the transition from epithelial to mesenchymal phenotype of metastatic cells (Warita et al., 2014). It was reported that statin supply is effective in reducing the occurrence of a wide range of cancers (e.g., liver, gastric, colorectal, pancreatic, and prostate) and cancer-related mortality ratios (Gbelcova et al., 2008; Ginestier et al., 2012; Nielsen et al., 2013; Benakanakere et al., 2014; Mok and Lee, 2020).

#### SREBP

Lipid homeostasis is controlled by membrane-bound transcription factors. These include the family of sterol regulatory element-binding proteins (SREBPs), which consist of three isoforms (SREBP1a, SREBP1c, and SREBP2). Among them, SREBP2 modulates more than 30 genes specific to the synthesis and uptake of cholesterol, fatty acids, triglycerides, and phospholipids (Horton et al., 2002; Luo et al., 2020). SREBPs are inactive when high cholesterol amounts are sensed and bound to SREBP cleavage activating protein (SCAP) and insulin-induced genes (INSIGs). Under cholesterol depletion conditions, feedback responses determine the dissociation of INSIGs from the SREBP2/SCAP complex, which move from the ER toward the Golgi apparatus. Subsequently, SREBP2 is processed and addressed to the nucleus. Nuclear SREBP2 (nSREBP2) induces the transcription of target genes, such as HMGCR and SQLE, by the direct interaction with the sterol regulatory element (SRE) motifs in their promoters (Brown et al., 2018). The upregulation of SREBP-activated downstream genes has been observed in a plethora of cancers, including glioblastoma (Guo et al., 2009; Yamauchi and Rogers, 2018), prostate cancer (Ettinger et al., 2004), breast cancer (Pitroda et al., 2009), and melanoma (Yamauchi et al., 2011; Kuzu et al., 2016), and was often found associated with poor prognosis/survival. The increase of cholesterol synthesis is mediated by the activation of PI3K–AKT–mTORC1 signaling (Porstmann et al., 2008; Duvel et al., 2010). In fact, PI3K–AKT pathway induced expression of SREBP genes and increased the stability of nuclear SREBPs by preventing their proteasomal degradation. Similarly, the transcription of nSREBP2 can also be induced by the mammalian target of rapamycin complex 1 (mTORC1) via inhibition of lipin-1 (Peterson et al., 2011; Mok and Lee, 2020). Results from preclinical studies showed that the AKT/mTORC1/SREBP pathway takes part in cancer cell growth by inducing biosynthesis of cholesterol (Porstmann et al., 2008). Finally, direct inhibition of SREBP2 (e.g., using fatostatin), resulting in a decrease in cholesterol content, may represent an interesting target in the treatment of a wide range of cancers (Gholkar et al., 2016; Gao et al., 2018).

#### SQLE

Squalene epoxidase catalyzes the conversion of squalene to (S)-2,3-epoxysqualene (SQLE) and shares with HMGCR the low rates of catalysis in the cholesterol biosynthesis. Like HMGCR, SQLE abundance and activity are regulated at gene and protein levels. The *SQLE* gene responds to sterols via SREBP2 (Nagai et al., 2002; Howe et al., 2017; Luo et al., 2020) and the protein can be degraded in the presence of cholesterol by ubiquitination. HMGCR and SQLE are both transcriptionally controlled by nSREBP2, but they can lead to cholesterol biosynthesis independently of each other. In the presence of an excess of cholesterol, squalene epoxidase is degraded by the E3 ubiquitin ligase MARCH6 (Yoshioka et al., 2020).

Robust scientific evidence demonstrated that SQLE can be considered an important oncogene and, consequently, may represent a possible target in cancer therapy (Cirmena et al., 2018). In particular, *in vitro* and *in vivo* studies have shown that SQLE could promote tumor cell proliferation and migration, and that treatment with its inhibitors (i.e., terbinafine or NB-598) could induce cancer cell demise (Chua et al., 2018). Moreover, a further study on nasopharyngeal carcinoma (NPC) linked the oncogenic effect of SQLE to the cholesteryl ester accumulation, which promoted NPC cell proliferation by activating the PI3K/AKT pathway (Li et al., 2020). In the same vein, in hepatocellular carcinoma (HCC), the overexpression of SQLE was demonstrated to promote cell proliferation and migration by ERK signaling, while downregulation of SQLE was able to inhibit the tumorigenicity of HCC cells (Sui et al., 2015). In addition, SQLE overexpression seem to represent a negative prognostic factor both in breast and prostate cancers (Stopsack et al., 2016; Brown et al., 2019).

#### NPC1L1

Niemann–Pick type C1-like 1 is the principal responsible for cholesterol absorption from dietary sources. It is a membrane protein expressed on the apical surface of enterocytes and on the membrane of bile canaliculi of human hepatocytes (Altmann et al., 2004). Under normal growth conditions, NPC1L1 is present primarily in the endocytic recycling compartment and translocate rapidly to the plasma membrane upon cholesterol depletion (Yu et al., 2006; Ge et al., 2008). Supplying of cholesterol triggers the inward transport of NPC1L1 together with cholesterol from the plasma membrane to the endocytic recycling compartment (Ge et al., 2008). NPC1L1 can be sent back to the plasma membrane for recycling. This process requires, in addition to other proteins, the small GTPase CDC42 and actin filaments (Zang et al., 2001; Xie et al., 2011; Luo et al., 2020). The human NPC1L1 gene is activated by SREBP2. Moreover, NPC1L1 protein amount can be modulated by degradation (Malhotra et al., 2019).

Recent literature data obtained both *in vivo* and *in vitro* indicated that NPC1L1 could represent a highly effective therapeutic target for treating pancreatic ductal adenocarcinoma (PDAC) because aberrant cholesterol uptake has been suggested to play a role in the proliferation and survival of pancreatic cancer cells (Guillaumond et al., 2015). Accordingly, the use of ezetimibe, an inhibitor of NPC1L1 clinically used for the hypercholesterolemia treatment, significantly reduced the survival capacity of PDAC cells (Nicolle et al., 2017) and tumor-associated blood vessel development (Solomon et al., 2009).

#### LDLR

Low-density lipoprotein receptor is a cell surface glycoprotein, which causes cellular absorption of cholesterol carried by the bloodstream. LDLR is the transcriptional target of SREBP2 and thyroid hormones (Lopez et al., 2007). LDLR binds circulating LDLs to clathrin-coated vesicles, which then accede the endocytic pathway (Goldstein and Brown, 2009). The endosome acidity induces conformational modifications that allow detaching LDLR from LDLs (Rudenko et al., 2002). LDLR is then redirected to the cell surface for further endocytic cycles (Bartuzi et al., 2016; Fedoseienko et al., 2018) or it is addressed to lysosomes for degradation. After endocytosis, lysosomal lipase hydrolyzes LDL-carried CE and produces cholesterol, which is finally exported by the coordinate actions of NPC2, NPC1, and LAMP2 (Kwon et al., 2009; Li and Pfeffer, 2016). Inhibition of the LDLR at any level (e.g., biosynthesis, membrane localization, internalization, recycling, and degradation) can affect its availability or functionality and, consequently, LDL removal. LXRs bind to promoter of a LDLR inhibitor (IDOL) and upregulate its expression (Zelcer et al., 2009). Accordingly, using synthetic LXR agonists decreased LDLR abundance and limited LDL uptake in cultured cells and livers of non-human primates (Zelcer et al., 2009; Luo et al., 2020). The increased PCSK9 binding with LDLR prevents LDLR recycling and promotes its degradation in lysosomes (Zhang et al., 2008; Chang et al., 2009; Luo et al., 2020). Several studies performed by using LXR agonists showed that cholesterol levels of glioblastoma cells decreased after stimulation of LXRs. The cholesterol decrement is due to enhanced cholesterol export and reduction of cholesterol import (Guo et al., 2011; Villa et al., 2016). LXR agonists also suppressed metastases of melanoma and prolonged animal survival in several *in vivo* models (Pencheva et al., 2014; Gabitova et al., 2015; Yamauchi and Rogers, 2018). In human cancers, the upregulation of PI3K–AKT–mTORC1 signaling promoted cellular cholesterol accumulation trough stimulation of LDLR-mediated cholesterol import. In glioblastoma, the overexpression of LDLR was caused by AKT, and pharmacological inhibition of LDLR actually favored cancer cell death (Guo et al., 2011; Kuzu et al., 2016). As mentioned previously, the increase of oxysterol, a by-product of cholesterol biosynthesis, was able to activate LXRs, which have been proposed as therapeutic targets in multiple cancer types (Lin et al., 2016; Komati et al., 2017).

#### SOAT

Sterol-*o*-acyltransferase (SOAT) is a protein of the rough surface ER responsible for cholesteryl ester (CE) production from cholesterol and long-chain fatty acids. SOAT plays an important function by regulating cellular cholesterol storage and free cholesterol content (Pramfalk et al., 2005). Because SOAT has a key role in regulating cellular cholesterol metabolism, it may represent a potential target for treatment of different sickness, including cancer (Rogers et al., 2015; Yang et al., 2020). For instance, it is well known that cholesterol esterification and lipid droplet formation are typical features of glioblastoma (GBM). In fact, accumulation of lipid droplets in the tumor in patients with glioma correlated with GBM progression and poor survival. Genetic inhibition of SOAT1 to block cholesterol esterification was observed to suppress GBM growth and prolong the survival in xenograft models (Geng et al., 2016). In addition, literature data also demonstrated that treating adrenocortical carcinoma (ACC) with mitotane reduced cancer cell survival by inhibiting SOAT1 activity (Sbiera et al., 2015). Very recently, Jiang and colleagues also highlighted that the high expression of SOAT1 was associated with a poor prognosis in hepatocellular carcinoma, whereas its inhibition suppressed cell proliferation and migration (Jiang et al., 2019).

#### Pyrophosphates

Different enzymes involved in the biosynthesis of cholesterol could be targeted by anticancer drugs. Farnesyl transferase (FTase) and geranylgeranyl transferases (GGTase) are enzymes that catalyzed the production of farnesyl pyrophosphate (FPP) and geranylgeranyl pyrophosphate (GGPP), both involved in cellular protein prenylation (Berndt et al., 2011; Sorrentino et al., 2014; Mok and Lee, 2020). The prenylation method consists in covalent bonds of hydrophobic molecules (both the C-15 isoprene farnesyl and the C-20 isoprene geranylgeranyl groups) to the C-terminal portion of different proteins, including the small GTP-binding proteins (sGTPases). sGTPases represent the greater and better described class of prenylated proteins. Each member of the Ras, Rho, and Rab GTPase families, many of which are involved in tumorigenesis, requires the structural alteration given by prenylation to bind to cell surface. Such posttranslational modifications and activation of GTP-binding proteins play a key function in most significant cellular processes. Inhibition of the mevalonate pathway reducing the isoprenylation of sGTPases could bring tumor cell death (Szajnman et al., 2005). Because the administration of GGPP and FPP prevented cancer cell death, it has been hypothesized that GGPP and FPP were necessary for the survival of cancer cells. It has been shown that bisphosphonates (BP) were able to block FPP synthase during the production of mevalonate, thus decreasing the formation of FPP and GGPP (Szajnman et al., 2005). Although BPs blocking osteoclast-mediated bone resorption are currently considered the most significant pharmaceutical treatment for metabolic bone disease, recently nitrogen-containing BPs have aroused interest in their antitumor properties (Nooh et al., 2017).

#### Steroid Hormones

Cholesterol is the precursor molecule of five different groups of steroid hormones synthesized by specific organs, the adrenal cortex and the gonads. Steroid hormones are widely known to control growth and differentiation. Moreover, steroid hormones are closely linked with the progression of ovarian, breast, and prostate cancers (Lukanova and Kaaks, 2005; Llaverias et al., 2011; Pelton et al., 2012; Murai, 2015; Radisauskas et al., 2016). According with this, inhibition of estrogen or androgen signaling represents the most used strategy in the treatment of these hormone-dependent tumors (Huang and Tindall, 2002; Lu-Yao et al., 2014; Fan et al., 2015; Watson et al., 2015; Simigdala et al., 2016; Shah et al., 2020). With regard to prostate cancer, although almost all patients with metastatic disease initially respond to androgen-ablation therapies, unfortunately the majority of patients progress to a castrate-resistant stage (Huang and Tindall, 2002; Lu-Yao et al., 2014; Watson et al., 2015; Shah et al., 2020). There is accumulating evidence suggesting that cholesterol, as a precursor of steroid hormones, effects on breast and prostate cancers. In this regard, it was observed that *in vitro* treatment of prostate and breast cancer cells with mevalonate was able to induce a high proliferation rate (Mokarram et al., 2017).

### Targeting Cholesterol Transport

To keep the cellular cholesterol levels constant, it is necessary that the excess cholesterol is carried out of the cell, and this can take place through active export and passive diffusion. Active export requires energy and involves several ABC transporter proteins using ATP. Passive diffusion is caused by a difference in cholesterol content between the cell membrane and cholesterol acceptors such as HDL.

#### LDLs/HDLs

Cholesterol is a highly insoluble molecule that is transported in the bloodstream via lipoproteins LDLs and HDLs. Cells can acquire exogenous cholesterol from LDLs that carry abundant cholesterol esters. LDL initially binds to the LDLR receptor on the plasma membrane and is subsequently internalized by clathrin vesicles (Brown and Goldstein, 1986). In LDL, the lipase activity degrades esterified cholesterol producing free cholesterol, which is internalized by the plasma membrane and reaches the endosome/lysosome (Sugii et al., 2003; Yamauchi et al., 2007; Yamauchi and Rogers, 2018). ApoA-I is one of the best known HDL apolipoproteins, capable of binding cholesterol and phospholipids. HDLs bind and carry excess cholesterol from peripheral tissues to the liver where cholesterol is transformed into bile acids for excretion (reverse cholesterol transport) (Yamauchi and Rogers, 2018). Lipoproteins are involved in the elaboration and transport of dietary cholesterol to tissues and aid in keeping a constant level of cholesterol by removing its excess to carry it to the liver. Therefore, lipoproteins could be involved in tumor progression by promoting the availability of cholesterol to tumor cells (Silvente-Poirot and Poirot, 2012; Cruz et al., 2013).

#### ABCA1

ATP-binding cassette transporters are drug efflux pumps that can cause multidrug resistance and tumor treatment failure (Deeley et al., 2006; Borel et al., 2012). Regarding ABCA1, it is responsible for the bidirectional transport of cholesterol across the cell surface, regulating its availability within the cell. In addition, it mediates the export of cholesterol and phospholipids in ApoA-I for HDL assembly (Oram and Heinecke, 2005). Some research works with *Abca1* knockout mice have shown that both hepatic and intestinal ABCA1 concur to the production of total plasma HDL, although to varying degrees (Timmins et al., 2005; Brunham et al., 2006). ABCA1 localizes both at the plasma membrane, where it mediates the assembly of nascent HDLs (Denis et al., 2008), and at the endocytic compartments, where it is degraded or recycled from the plasmatic membrane (Lu et al., 2008). An excess of ABCA1 generates deformation sites in the plasma membrane, where apoA-I binds (Vedhachalam et al., 2007). This could be a result of the disturbing action of ABCA1 on lipid raft organization (Landry et al., 2006). In contrast, ABCA1 deficiency has been found to increase lipid rafts and cholesterol contents in the plasma membrane (Koseki et al., 2007; Zhu et al., 2008; Yamauchi et al., 2015). ABCA1 is closely regulated both at gene and protein levels. LXRs are mainly responsible for activating the *ABCA1* gene (Costet et al., 2000; Venkateswaran et al., 2000). Accordingly, data obtained *in vitro* by using LXR agonists revealed that the activation of LXR reduced intracellular cholesterol amount by increasing its exportation and decreasing its absorption therefore determining a growth inhibition of glioblastoma cells (Guo et al., 2011; Villa et al., 2016). Moreover, LXR agonists suppressed melanoma metastases and prolonged animal survival in several *in vivo* models (Pencheva et al., 2014; Gabitova et al., 2015; Yamauchi and Rogers, 2018).

On the other hand, it was also reported the PI3K/AKT signaling, through the inhibition of ABCA1 mTORC1-dependent pathway, resulted in an increase of intracellular level of cholesterol (Porstmann et al., 2008; Dong et al., 2014). Rapamycin, the mTORC1 inhibitor, repressed the cholesterol export due to the action of ABCA1 (Dong et al., 2014).

Finally, the regulation of *ABCA1* gene was also modulated by some miRNAs, including miR-33a/b and miR-148 (Najafi-Shoushtari et al., 2010; Rayner et al., 2011; Feinberg and Moore, 2016). These miRNAs suppressed *ABCA1* gene expression and reduced plasma HDL levels in mice and non-human primates (Najafi-Shoushtari et al., 2010; Yamauchi and Rogers, 2018).

#### ACAT

Acetyl-CoA acetyltransferase 1 and 2 (ACAT1 and ACAT2, respectively) play an important role in lipid metabolism. After esterification into CEs by ACAT1, surplus cholesterol can be stored as lipid droplets (Chang et al., 2009). ACAT1 overexpression and CE accumulation were found to play pro-tumor functions. In fact, it was observed that ACAT1 induced an excessive storage of cholesterol in lipid droplets in prostate (Yue et al., 2014), pancreatic cancers (Li et al., 2016), and glioblastoma (Geng et al., 2016). According with this, ACAT1 inhibition was found to reduce migration of breast cancer cells (Antalis et al., 2010) and decrease prostate cancer progression (Saraon et al., 2014). Moreover, ACAT1 inactivation determined abundant ER cholesterol content, which is acting on SREBP-1 reduced cholesterol synthesis and uptake (Yue et al., 2014). Recently, pharmacological or genetic ACAT1 inhibition has been associated with attenuated cancer cell proliferation (Yang et al., 2020).

In light of all this, ACAT1 can be considered a possible therapeutic target for the treatment of some neoplasms linked to the abundant presence of cholesterol esters. The use of avasimibe, an effective inhibitor of ACAT1, remarkably decreased cholesteryl esters stocked in lipid droplets and enhanced free cholesterol amount into the cell in human prostate, pancreatic, lung, glioblastoma, and colon cancer cells leading to inhibition of proliferation, cell cycle arrest, and apoptosis (Yang et al., 2020).

## Conclusion

As the metabolic needs of cancer cells appear significantly different from non-cancer cells, the metabolic pathways could represent potential innovative targets for a more selective anticancer therapy.

The analysis of literature data confirmed a fundamental role of cholesterol and its metabolism in many and different human pathologies, including atherosclerosis, Alzheimer’s disease, and other neurodegenerative disorders, and mounting evidence connected cholesterol homeostasis disorder and cancer. Moreover, dysregulation of the mevalonate pathway has been found to promote cell transformation, thus suggesting the role of oncogene for *HMG-CoA reductase* (Clendening et al., 2010). All of this makes cholesterol a potential attractive pharmacological target also in oncology context. Accordingly, although quite controversial, preclinical and epidemiological data seem to show an antitumor activity of statins, at least against some types of cancer (Solomon and Freeman, 2008).

At present, several drugs targeting cholesterol metabolism besides statins are clinically used to inhibit cholesterol production and increase LDL absorption, to hinder NPC1L1 absorption of dietary cholesterol and LDLR degradation, and to reduce plasma LDLs. For instance, the cholesterol uptake-blocking agent ezetimibe, by decreasing circulating cholesterol levels, has been found to reduce prostate tumor growth (Solomon et al., 2009).

Moreover, an increasing number of preclinical and clinical studies are underway to define as new potential therapeutic target for cancer therapy SREBP and its regulators LXRs, the transporters ABCs, and ACATs.

Increasing evidence showed that also cholesterol-rich raft membrane domains could represent a promising target in cancer therapy (Mollinedo and Gajate, 2006, 2020; Mollinedo et al., 2010a). Indeed, acting as scaffolds for signaling pathways in the cell membrane, lipid rafts promote and facilitate the triggering of both survival and apoptosis by recruiting signaling molecules and death receptors. In this regard, understanding how the different rafts that promote cell survival or death are temporarily formed and disrupted may be an important goal to understand if and how lipid rafts could represent a specific target in cancer therapy.

Finally, it is important to underline the key role played by the intestinal microbiota in the reduction and elimination of cholesterol, thus contributing significantly to its homeostasis (Zimber et al., 2000; Antharam et al., 2016). An alteration of the bacterial flora could constitute a further risk factor, especially for those tumors particularly dependent on cholesterol homeostasis. Thus, acting on microbiota might also represent a further possibility of therapeutic intervention in the treatment and/or prevention of certain types of cancer.

## Author Contributions

RV contributed to conceive the idea, drafted the article, and drew figures relative to cholesterol metabolism. EI contributed to design and to draft the article and drew figures relative to lipid rafts. PM conceived the idea, draft and revised the article, coordinated the activity, and concurred to the final version of the review. All authors contributed to the article and approved the submitted version.

## Conflict of Interest

The authors declare that the research was conducted in the absence of any commercial or financial relationships that could be construed as a potential conflict of interest.

## Abbreviations

ABCA1: ATP-binding cassette subfamily A member 1
ABCG1: ATP-binding cassette subfamily G member 1
ACAT1: acyl-CoA:cholesteryl acyltransferase 1
AMPK: 5 ′ adenosine monophosphate-activated protein kinase
AMPK: AMP-activated protein kinase
ApoA-I: lipid-poor apolipoprotein A-I
CE: cholesteryl ester
ER: endoplasmic reticulum
FPP: farnesyl pyrophosphate
FPP: farnesyl pyrophosphate
FTase: farnesyl transferase
GGPP: geranylgeranyl pyrophosphate. GGTase, geranylgeranyl transferases
HDL: high-density lipoprotein
HMG-CoA: 3-hydroxy-3-methyl-glutaryl CoA
HMGR: HMG reductase
IGF: insulin-like growth factor
INSIGs: insulin-induced gene
LDL: low-density lipoprotein
LDLR: LDL receptor
LXR: liver X receptor
M β CD: methyl- β-cyclodextrin
miRNA: microRNA
mTOR: mammalian target of rapamycin
mTORC1: mammalian target of rapamycin complex 1
NPC1L1: Niemann–Pick type C1-like 1
PI3K: phosphatidylinositol 3-kinase
SCAP: SREBP cleavage activating protein
sGTPase: small GTP-binding protein
SOAT: sterol-o-acyltransferase
SQL: squalene
SQLE: squalene epoxidase
SRE: sterol regulatory element
SREBP2: sterol regulatory element-binding protein 2
VLDL: very-low-density lipoprotein.
